# Effects of Anthocyanin-Rich Sorghum Bran Extract on the Quality, Antioxidant Stability, Processing Safety, and Flavor of Taosu—A Chinese Shortbread Cookie

**DOI:** 10.3390/foods15142444

**Published:** 2026-07-09

**Authors:** Shitao Xiong, Kanxin Ye, Miao Liang, Ping Zhang, Yousheng Huang, Leiyan Wu, Hua Zhang, Yun Xiong

**Affiliations:** 1 Key Laboratory of Modern Preparation of Chinese Medicine, Department of Development and Evaluation of Dietary Functional Products, Ministry of Education, Jiangxi University of Chinese Medicine, Nanchang 330004, China; xiongshitao@jxutcm.edu.cn (S.X.); yekanxin@jxutcm.edu.cn (K.Y.); liangmiao@jxutcm.edu.cn (M.L.); zhangping2@jxutcm.edu.cn (P.Z.); yshuang0526@163.com (Y.H.); 2 College of Food Science and Engineering, Jiangxi Agricultural University, Nanchang 330045, China; fswuly@jxau.edu.cn

**Keywords:** sorghum bran extract, anthocyanins, 3-deoxyanthocyanidins, Taosu, antioxidant stability, acrylamide, 5-HMF, flavor profile

## Abstract

Sorghum bran, an abundant milling by-product, is rich in phenolics but underused as a food ingredient. Anthocyanin-rich sorghum bran extract (SBE) was added to Taosu, a traditional Chinese shortbread cookie, at 0–2%, and its effects on quality, flavor, antioxidant stability during storage, and heat-induced contaminants were evaluated. UHPLC-QTOF-MS/MS showed that SBE was rich in flavones (apigenin, luteolin) and 3-deoxyanthocyanidin apigeninidin and had strong in vitro antioxidant capacity; these phenolics transferred dose-dependently into Taosu. SBE raised the total phenolic, flavonoid, and anthocyanin contents and the radical-scavenging capacity and imparted a natural reddish color. The instrumental taste profile was essentially unchanged, whereas HS-SPME-GC-MS revealed a dose-dependent shift in the volatile profile, with more Maillard-derived furans (e.g., furfuryl alcohol) and fewer lipid-oxidation aldehydes at the highest level. At the 2% level, acrylamide and 5-hydroxymethylfurfural (5-HMF) were reduced by 30.9% and 46.0%, respectively. After 14 days of storage, the fortified cookies retained much higher phenolic and antioxidant levels than the control, with the 2% sample still exceeding the fresh control, indicating improved retention of phenolics and antioxidant capacity during the period evaluated. Overall, sorghum bran offers a route to upcycle a low-value by-product into a clean-label, multifunctional ingredient that improves the healthfulness and processing safety of traditional baked goods.

## 1. Introduction

Taosu is a traditional Chinese shortbread cookie with a long history, characterized by a crumbly tender texture, a rich baked aroma, and a golden-brown color, and it occupies an important position in the Chinese pastry market. It is typically formulated with wheat flour, fat, sugar, and small amounts of leavening agents, and is produced by high-temperature baking; the final fat content of Taosu is generally above 25–35% with a low moisture content [1]. Such a high-fat and low-moisture matrix imparts the distinctive crispness and baked flavor of Taosu, but it also makes the product prone to lipid oxidation during processing and storage, generating primary and secondary oxidation products such as hydroperoxides, aldehydes, and malondialdehyde, which lead to flavor deterioration, textural changes, and shortened shelf life [2,3,4,5]. Therefore, improving the oxidative stability and storage quality of Taosu represents a practically meaningful direction in the research of such traditional pastries.

With the growing popularity of the “clean-label” concept and increasing consumer concerns about synthetic additives, the use of natural plant-derived functional ingredients in bakery products has received considerable attention [6]. Plant polyphenols and anthocyanins, owing to their multiple functions including natural pigmentation, free radical scavenging, metal-ion chelation, and inhibition of lipid oxidation, have been incorporated into various bakery products, such as bread, cookies, and muffins [7,8,9], offering new possibilities for the development of bakery products that combine functional and natural-coloring properties. Beyond oxidation, the high-temperature baking of such products also promotes Maillard and caramelization reactions that generate heat-induced contaminants, notably acrylamide and 5-hydroxymethylfurfural (5-HMF), which are of increasing food-safety concern; plant phenolics have been reported to suppress the formation of these contaminants, providing a further incentive for their incorporation into baked goods [10,11,12].

Sorghum (*Sorghum bicolor* L. Moench) is an important coarse cereal crop worldwide, and its bran, generated as a by-product during processing, is enriched in most of the polyphenols, flavonoids, and pigment-related bioactive compounds present in the kernel, representing a high-quality source of natural functional components [13,14]. Global sorghum production is about 64 million tons per year [15], and the bran removed during milling and decortication represents roughly 8–10% of the grain, so a large tonnage of this low-value by-product is generated annually. Among sorghum phenolics, 3-deoxyanthocyanidins (3-DXAs), such as apigeninidin and luteolinidin, are major bioactive components and characteristic red pigments of sorghum. Unlike conventional anthocyanins, which are readily degraded by heat, 3-DXAs lack the C-3 hydroxyl group and are therefore much more stable to heat and pH, as well as being strong radical scavengers [13,14,16]. In addition to 3-DXAs, sorghum bran extracts also contain proanthocyanidins, flavonols, and phenolic acids, exhibiting an overall favorable antioxidant potential [13]. These characteristics give anthocyanin-rich sorghum bran extract (SBE) clear potential for the development of functional bakery products, as supported by recent applications in wheat-based bread and Chinese steamed bread [10,17], providing a practical pathway for the value-added utilization of sorghum by-products. However, such work has so far concentrated on wheat bread and steamed bread, and the behavior of SBE in high-fat, low-moisture baked products has received little attention.

Taosu is a typical product of this kind, in which both oxidative stability and thermal-processing safety are at stake, and was therefore selected as the model food in this study. Although whole sorghum flour is increasingly used in baked goods, sorghum bran, a phenolic-rich by-product [18], and especially its extract have rarely been incorporated into bakery products [10], and its effect in a high-fat, low-moisture shortbread such as Taosu, where lipid oxidation rather than staling governs shelf life, has not been reported. To address this gap, the present study systematically evaluated the effects of anthocyanin-rich SBE in Taosu. Specifically, SBE was incorporated into Taosu formulation at four levels (0%, 0.5%, 1%, and 2%), and the following aspects were comparatively evaluated: (1) the major phenolic and anthocyanin composition of SBE and its in vitro antioxidant capacity; (2) the effects of SBE addition on basic quality attributes of Taosu, including color, moisture, and texture; (3) the retention of bioactive compounds and antioxidant capacity of Taosu during storage; (4) the effect of SBE on the formation of acrylamide and 5-HMF during baking; and (5) the effect of SBE on the taste characteristics and volatile flavor profile of Taosu. The findings are expected to provide a reference for the value-added utilization of sorghum bran by-products and for the functional upgrading of traditional Chinese bakery products.

## 2. Materials and Methods

### 2.1. Materials and Chemicals

SBE was obtained from Shandong Zhonghui Biotechnology Co., Ltd. (Binzhou, Shandong, China). Low-gluten wheat flour, unsalted butter, corn oil, powdered sugar, sodium bicarbonate, and baking powder were purchased from commercial suppliers in China. SBE was prepared from red sorghum bran by aqueous–ethanol extraction and supplied as a food-grade powder (pH 7.3; loss on drying 4.73%; arsenic and lead were below limits of detection).

Gallic acid; rutin; luteolinidin chloride; Trolox; acrylamide (AA); 5-hydroxymethylfurfural (5-HMF); 13C3-AA; 13C6-5-HMF; 3-octanone; C6–C40 n-alkanes; Folin–Ciocalteu reagent; 2,2′-azino-bis(3-ethylbenzothiazoline-6-sulfonic acid) diammonium salt (ABTS); 2,2-diphenyl-1-picrylhydrazyl (DPPH); 2,4,6-tripyridyl-s-triazine (TPTZ); and all other reagents and chemicals were of analytical grade or higher unless otherwise stated and purchased from Shanghai Macklin Biochemical Co., Ltd. (Shanghai, China); Wuhan Tianzhi Biotechnology Co., Ltd. (Wuhan, China); or Sigma-Aldrich, Merck KGaA (Darmstadt, Germany).

### 2.2. Preparation of Taosu Samples and Storage Conditions

**Figure 1:**
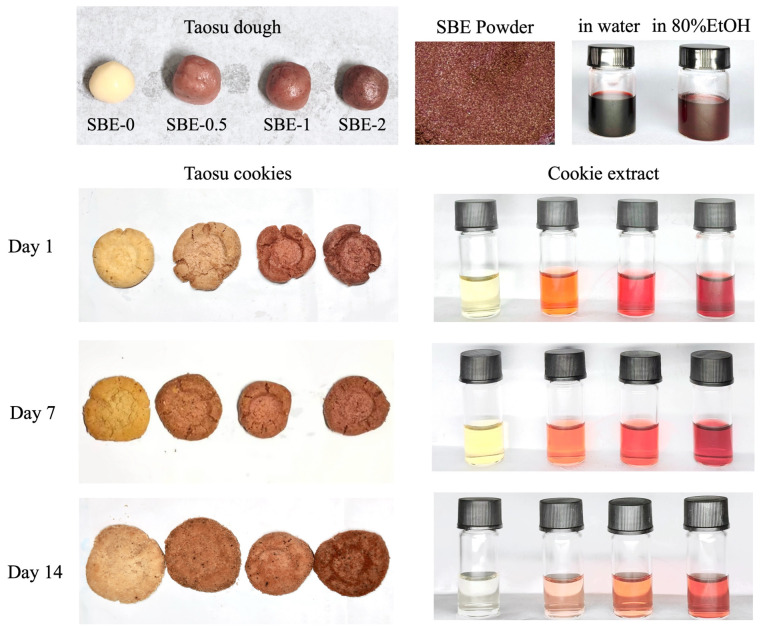
Appearances of the SBE and Taosu samples. (**Top**): Taosu dough prepared with SBE-0, SBE-0.5, SBE-1, and SBE-2, and the SBE powder with its color in water and in 80% ethanol. (**Bottom**): Baked Taosu cookies and the corresponding 80% ethanol cookie extracts on storage days 1, 7, and 14. In each multi-sample image, from left to right: SBE-0, SBE-0.5, SBE-1, and SBE-2 (0, 0.5, 1, and 2% SBE).

Taosu samples were prepared using the formulation shown in Table 1. SBE was incorporated at 0%, 0.5%, 1.0%, and 2.0% (SBE-0, SBE-0.5, SBE-1, and SBE-2, respectively) of the total dry powder weight by replacing an equivalent amount of low-gluten wheat flour, while the amounts of other ingredients were kept constant. Briefly, softened butter, corn oil, powdered sugar, sodium bicarbonate, and baking powder were mixed, followed by the addition of sieved SBE and low-gluten wheat flour. The dough was gently mixed, divided into 20.0 ± 0.5 g portions, hand-shaped and standardized in diameter and thickness with a ruler, and baked in a hot-air convection oven at 170 °C for 17 min. Each formulation was prepared as three independent batches (separate mixing, dough portioning, and baking). After cooling, the samples were divided into two sets according to their intended use. For the shelf-life study, Taosu samples were placed in open (unsealed) aluminum-foil resealable bags, stored at 25 ± 5 °C and about 80% relative humidity, and then sampled on days 1, 7, and 14; samples for all other analyses were freshly prepared. The appearances of the SBE, dough, baked Taosu, and corresponding cookie extracts are shown in Figure 1.

### 2.3. UHPLC-QTOF-MS/MS Analysis of Phenolic and Anthocyanin Compounds in SBE and Taosu

Extraction and UHPLC-QTOF-MS/MS analysis were performed with modifications based on previous studies [14,19]. UHPLC-QTOF-MS/MS analysis was performed using a UHPLC system coupled to a TripleTOF™ 6600 quadrupole time-of-flight mass spectrometer (SCIEX, Framingham, MA, USA). Chromatographic separation was achieved on a MicroPulite XP T3 column (2.1 × 100 mm, 3.5 μm, WePure Biotech, Guangzhou, Guangdong, China). The mobile phase consisted of water containing 0.1% formic acid as solvent A and acetonitrile containing 0.1% formic acid as solvent B. The flow rate was 0.30 mL/min, the column temperature was maintained at 35 °C, and the autosampler temperature was set at 4 °C. The gradient elution program was as follows: 0–2 min, 3% B; 2–8 min, 3–15% B; 8–16 min, 15–30% B; 16–21 min, 30–50% B; 21–26 min, 50–70% B; 26–28 min, 70–95% B; 28–30 min, 95% B; 30–30.5 min, 95–3% B; and 30.5–34 min, 3% B.

Mass spectrometric data were acquired in both positive and negative electrospray ionization modes using TOF MS and information-dependent acquisition MS/MS. The TOF MS scan range was *m*/*z* 100–1500, and the MS/MS scan range was *m*/*z* 50–1500. The ion source parameters were as follows: ion spray voltage, +5500 V in positive mode and −4500 V in negative mode; source temperature, 500 °C; curtain gas, 30 psi; ion source gas 1, 50 psi; ion source gas 2, 50 psi; declustering potential, ±80 V; collision energy, ±35 eV; and collision energy spread, ±15 eV. Spectral data were processed in MS-DIAL (v5.5.260323) and MS-FINDER (v3.73), and compounds were tentatively identified (Table 2) by matching accurate mass and MS/MS fragmentation against the MSP spectral library (v2024.08), with mass errors generally within ±5 ppm.

### 2.4. Analysis of Moisture, Texture, and Color Properties

Moisture content was determined according to the AOAC oven-drying method. Briefly, approximately 4.0 g of a ground Taosu sample was dried at 105 °C to a constant weight, and the moisture content was calculated based on the weight loss after drying.

Texture properties were determined using a TA.XTplus texture analyzer (Stable Micro Systems, Surrey, UK) equipped with a P/75 cylindrical probe, with modifications based on previously reported methods [20]. Baked Taosu samples were analyzed using a compression mode under the following conditions: pre-test speed, 5.0 mm/s; test speed, 2.0 mm/s; post-test speed, 5.0 mm/s; trigger force, 5.0 g; strain, 50%; and interval time, 5 s. Hardness and fracturability were used as the main texture indicators, while other TPA-related parameters were recorded as supplementary information.

Color parameters were measured using a CR-400 chroma meter (Konica Minolta, Tokyo, Japan) after calibration with the standard white plate supplied with the instrument. For each group, nine independent Taosu samples were analyzed (*n* = 9), and three different positions were measured on each sample, including the center and peripheral regions. The L*, a*, and b* values were recorded, the total color difference (ΔE) was calculated relative to the SBE-0 control, and average values were used for analysis (*n* = 27).

### 2.5. Analysis of Phenolic Contents and Antioxidant Capacity

The bioactive compound contents and antioxidant capacity of Taosu samples were determined using ethanol extracts. Briefly, 2.0 g of a ground Taosu sample was extracted with 5 mL of 80% ethanol by ultrasonication for 20 min, followed by centrifugation at 8000× *g* for 15 min. The residue was re-extracted twice with 2 mL of 80% ethanol, and the combined supernatants were used for subsequent analyses.

The total phenolic content (TPC), total flavonoid content (TFC), and total anthocyanin content (TAC) were determined using a Spark 20M multimode microplate reader (Tecan, Männedorf, Switzerland) based on previously reported methods [21,22]. TPC was determined using the Folin–Ciocalteu method, with gallic acid as the standard, and the results were expressed as mg gallic acid equivalents per gram of sample (mg GAE/g). TFC was measured using the aluminum chloride colorimetric method, with rutin as the standard, and the results were expressed as mg rutin equivalents per gram of sample (mg RE/g). TAC was determined using the pH differential method [22], with pH 1.0 and pH 4.5 buffer solutions, and measured at 520 nm (with 700 nm background subtraction). Luteolinidin was used as the standard, and the results were expressed as mg luteolinidin equivalents per gram of sample (mg LutE/g).

The antioxidant capacity of Taosu extracts was evaluated using the 2,2′-azino-bis(3-ethylbenzothiazoline-6-sulfonic acid) (ABTS) radical scavenging assay, 2,2-diphenyl-1-picrylhydrazyl (DPPH) radical scavenging assay, and ferric reducing antioxidant power (FRAP) assay based on previously reported methods [21]. For the ABTS and DPPH assays, the corresponding radical working solutions were mixed with sample extract or Trolox standard solution and incubated in the dark at room temperature for 30 min before absorbance measurement at 734 nm and 517 nm, respectively. For the FRAP assay, freshly prepared FRAP working solution was mixed with sample extract or Trolox standard solution, incubated at 37 °C for 30 min, and measured at 593 nm. Trolox was used as the standard, and the results were expressed as mg Trolox equivalents per gram of sample (mg TE/g). Calibration parameters for the six spectrophotometric assays (standard, linear range, regression equation, and R^2^) are provided in Table S4.

### 2.6. Determination of Acrylamide and 5-HMF by LC-MS/MS

AA and 5-HMF were determined by LC-MS/MS based on a previously reported method [23]. A ground Taosu sample (1.0 g) was spiked with 250 μL of mixed isotope-labeled internal standard solution, mixed with 3 mL of ultrapure water, and extracted with 10 mL of acetonitrile. After adding 1.0 g NaCl and 4.0 g anhydrous MgSO_4_, the mixture was shaken for 20 min and centrifuged at 4400× *g* for 5 min. The supernatant (5 mL) was dried under nitrogen at 40 °C, reconstituted in 1.0 mL of distilled water, filtered through a 0.22 μm aqueous membrane, and analyzed. Method validation parameters (linearity, LOD, LOQ, recovery, and precision) are given in Table S5.

LC-MS/MS analysis was performed using a Shimadzu LC-30AD UHPLC system equipped with a DGU-20A5R degassing unit and a SIL-30AC autosampler (Shimadzu Corporation, Kyoto, Japan) and coupled to a Triple Quad 5500 mass spectrometer (SCIEX, Framingham, MA, USA). Separation was achieved on a Poroshell 120 Aq-C18 column (2.1 × 100 mm, 2.7 μm; Agilent Technologies, Santa Clara, CA, USA) using acetonitrile and 0.1% formic acid in water as mobile phases at a flow rate of 0.30 mL/min, with the column temperature set at 30 °C and the injection volume set at 5.0 μL. The gradient was 5–80% B from 0 to 1 min, maintained at 80% B from 1 to 3.1 min, returned to 5% B from 3.1 to 4 min, and re-equilibrated to 6 min. Quantification was performed in positive ESI-MRM mode using ^13^C_3_-AA and ^13^C_6_-5-HMF as internal standards. The transitions *m*/*z* 72 → 55 and *m*/*z* 127 → 53 were used for quantification of AA and 5-HMF, respectively, while *m*/*z* 72 → 44 and *m*/*z* 127 → 81 were used for confirmation. Calibration curves were prepared using mixed standard solutions at 5–200 ng/mL, and results were expressed as μg/kg sample.

### 2.7. Electronic Tongue Analysis

The taste profile of Taosu samples was analyzed using a Taste Sensing System SA402B Plus electronic tongue (Insent Inc., Atsugi, Japan). A ground sample (0.25 g) was mixed with 50 mL of ultrapure water, stirred for 1 min, ultrasonically extracted for 15 min, centrifuged at 8000× *g* for 15 min, and filtered before analysis. The recorded taste attributes included sourness, bitterness, astringency, aftertaste-B, aftertaste-A, umami, richness, and saltiness. Each sample was analyzed in triplicate (*n* = 3). The taste sensors were calibrated and their stability verified against the reference solution before each measurement.

### 2.8. HS-SPME-GC-MS Analysis of Volatile Compounds

Volatile compounds in Taosu samples were analyzed by headspace solid-phase microextraction coupled with gas chromatography–mass spectrometry (HS-SPME-GC-MS) based on published methods [24,25]. The samples were cryogenically ground, passed through a 40–60 mesh sieve, and homogenized. Ground samples (3.0 g) were placed into a 20 mL amber headspace vial, followed by the addition of 5 μL of 3-octanone internal standard. The vial was sealed with a PTFE/silicone septum and equilibrated at 50 °C for 20 min with agitation at 350 rpm. Volatile compounds were extracted at 50 °C for 50 min using a PAL Smart SPME Arrow fiber (DVB/PDMS, 120 μm, 1.1 mm; Restek, Bellefonte, PA, USA), followed by thermal desorption at 250 °C for 5 min in splitless mode.

GC-MS analysis was performed using an Agilent 8890 gas chromatograph coupled to an Agilent 7000D mass spectrometer (Agilent Technologies, Santa Clara, CA, USA). Separation was achieved on an HP-5MS UI column (30 m × 0.25 mm × 0.25 μm; Agilent Technologies, Santa Clara, CA, USA). Helium was used as the carrier gas at a constant flow rate of 1.0 mL/min. The oven temperature was held at 40 °C for 3 min, increased to 180 °C at 3 °C/min, and then increased to 250 °C at 4 °C/min and held for 5 min. The MS was operated in electron ionization mode at 70 eV, with an ion source temperature of 230 °C, transfer line temperature of 250 °C, and scan range of *m*/*z* 35–450.

Volatile compounds were tentatively identified by comparing their mass spectra with the NIST23 library (reverse match factor ≥ 70) and by comparing retention indices, calculated using C6–C40 n-alkanes, with the literature’s values. Relative quantification was performed using the internal standard method, and the results were expressed as the ratio of the peak area of each compound to that of the internal standard.

### 2.9. Statistical Analysis

All experiments were performed at least in triplicate (*n* = 3) unless otherwise stated, and the results were expressed as mean ± standard deviation (SD). Statistical analysis was performed using IBM SPSS Statistics 26.0 (IBM Corp., Armonk, NY, USA). Before analysis of variance, the normality of residuals and the homogeneity of variances were verified using the Shapiro–Wilk and Levene tests, respectively. Differences between groups were analyzed by one-way analysis of variance (ANOVA), followed by Tukey’s honestly significant difference (HSD) test, with *p* < 0.05 considered statistically significant.

Pearson correlation analysis was used to evaluate relationships among the measured parameters; these correlation analyses, including the comprehensive matrix in Supplementary Table S6, were performed in RStudio 2026.04.0 (Posit Software, PBC, Boston, MA, USA). Principal component analysis (PCA), partial least squares (PLS) regression against SBE dose, heatmap analysis, hierarchical clustering, radar charts, and other graphical visualizations were performed using RStudio 2026.04.0 (Posit Software, PBC, Boston, MA, USA) and OriginPro 2026 (OriginLab Corporation, Northampton, MA, USA).

## 3. Results and Discussion

### 3.1. Composition and Antioxidant Characteristics of Sorghum Bran Extract

To establish the compositional basis for the application study, the phenolic profile of SBE was characterized by UHPLC-QTOF-MS/MS in both positive and negative ionization modes, and the same compounds were monitored in the Taosu extracts. A total of 89 phenolic compounds were tentatively identified (Table 2; full data with peak areas are shown in Table S1) by matching accurate mass and MS/MS fragmentation against the database, with mass errors generally within ±5 ppm (these tentative identifications correspond to Schymanski confidence level 2: accurate mass (≤5 ppm), diagnostic MS/MS key fragment ions, spectral library matching, and retention time); the complete peak-area data are provided in the Supplementary Materials. The identified compounds spanned seven classes: flavones (44), hydroxycinnamic acids (11), flavanones (9), anthocyanins (7), flavonols (7), isoflavones (7), and stilbenes (4), confirming that sorghum bran is a structurally diverse source of polyphenols.

Among the anthocyanins, the 3-deoxyanthocyanidin apigeninidin was the predominant pigment, clearly exceeding the conventional 3-hydroxyanthocyanins detected (pelargonin and the malvidin, cyanidin and peonidin glucosides). The flavanone precursors of the 3-deoxyanthocyanidin pathway, naringenin and eriodictyol, were also present, with naringenin being markedly more abundant [16]. Unlike conventional anthocyanins, 3-deoxyanthocyanidins lack the hydroxyl group at the C-3 position, which confers superior stability over a wide pH range and at the elevated temperatures encountered during baking, making them particularly attractive as natural colorants and functional ingredients for cereal foods [16]. The intense red color of the SBE powder and of its ethanolic solution (Figure 1) is consistent with this 3-deoxyanthocyanidin-rich profile, as apigeninidin and related 3-deoxyanthocyanidins are characteristic orange–red pigments [26].

In addition, SBE contained flavonols (e.g., kaempferol), isoflavones (e.g., daidzein, glycitein and biochanin A), hydroxycinnamic acids (e.g., dihydroferulic and caffeoylquinic acids), and stilbenes (resveratrol and pterostilbene), indicating that its antioxidant potential derives from a broad spectrum of phenolic structures rather than from a single dominant compound [13].

Consistent with this phenolic richness, SBE exhibited high contents of total phenolics (11.234 ± 0.147 mg GAE/g), total flavonoids (18.264 ± 0.786 mg RE/g), and total anthocyanins (7.778 ± 0.251 mg LutE/g), together with strong in vitro antioxidant capacity (ABTS, 6.583 ± 0.302; DPPH, 3.650 ± 0.244; FRAP, 5.497 ± 0.146 mg TE/g). These results confirm that the extract is a concentrated reservoir of antioxidant phenolics and provide the chemical basis for the functional effects observed in SBE-fortified Taosu [21,22,26,27].

**Figure 2:**
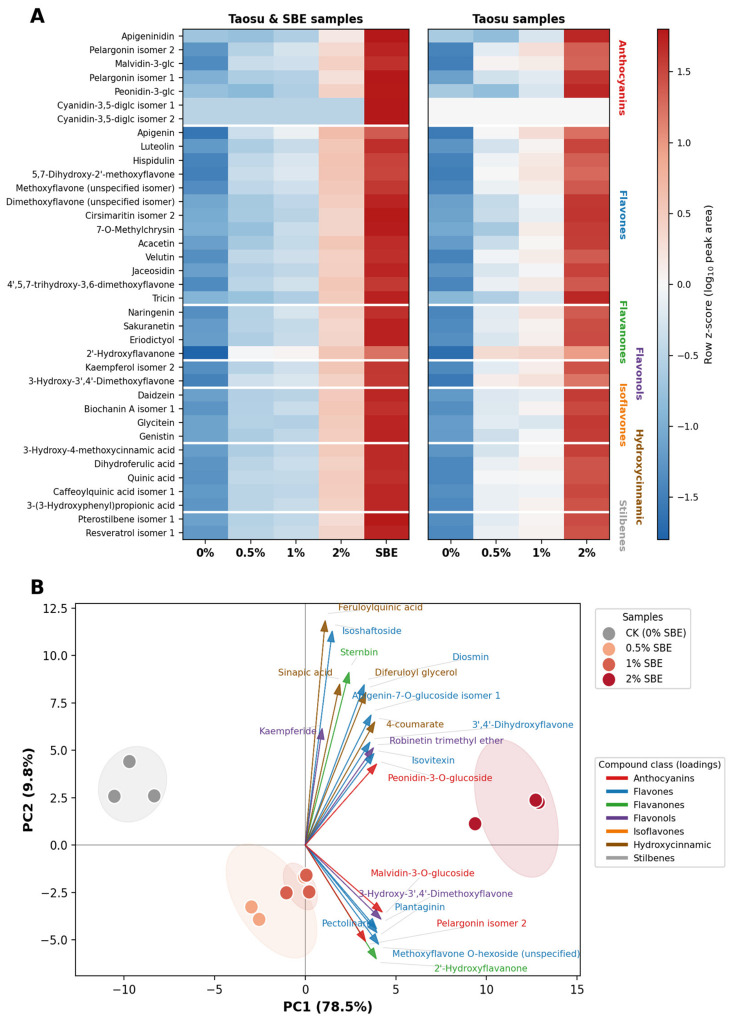
(**A**) Heatmap of representative phenolic compounds in SBE-fortified Taosu, shown with the SBE (**left**) and four Taosu formulations (**right**) (row-wise z-score of log10 peak area). (**B**) Principal component analysis of the phenolic profiles of four Taosu formulations (*n* = 3 per group; phenolic compounds; loadings colored by compound class).

When incorporated into Taosu, the majority of these SBE-derived phenolics increased in a dose-dependent manner from the control to the 2% level, approaching the profile of the extract itself (Figure 2A). Principal component analysis of the phenolic profiles (Figure 2B; PC1 = 75.4%, PC2 = 10.2%) separated the formulations along PC1 according to the SBE level, indicating that the characteristic phenolic and 3-deoxyanthocyanidin signature of SBE was largely retained after baking and thus remained available to exert antioxidant and related functional effects in the product.

### 3.2. Color, Moisture, and Texture Properties of Taosu

SBE addition altered the color of Taosu in a clear dose-dependent manner (Table 3). Lightness (L*) decreased progressively from 46.31 in the control to 28.28 at the 2% level, redness (a*) increased from 5.14 to 12.20, and yellowness (b*) declined from 18.93 to 9.64. Each of these three color parameters differed significantly among the formulations (*p* < 0.05), showing progressive darkening and antioxidant gain with anthocyanin-rich additions, a pattern consistent with recent cookie studies [28]. This darkening and shift toward red are largely attributable to the red-hued anthocyanins, 3-deoxyanthocyanidin and flavonoid pigments of SBE, giving the cookies an increasingly deep reddish-brown appearance. The shifts in L*, a*, and b* broadly scaled with the amount of 3-deoxyanthocyanidin (mainly apigeninidin) and flavonoid pigments incorporated from SBE (Table 2, Figure 2), suggesting that the coloration arose mainly from pigment incorporation rather than from baking-induced browning, and that SBE could serve as a natural colorant for Taosu. Comparable dose-dependent darkening and increases in redness have been reported for biscuits and shortbread cookies fortified with anthocyanin- or polyphenol-rich plant materials [4,8]. Notably, this color was a darker, reddish-brown (chocolate-like) tone rather than a bright red, reflecting the combined contribution of the SBE-derived pigments and Maillard browning during baking. This dose-dependent darkening and reddening was also clearly visible in both the dough and the baked cookies (Figure 1). Notably, this red coloration remained largely stable through baking rather than being lost, which can be attributed mainly to the high thermal stability of the apigeninidin-type 3-deoxyanthocyanidins that predominate in SBE, although proanthocyanidins and other phenolic pigments carried into the cookies are likely to contribute as well [13,16].

Moisture content decreased with SBE addition, from 1.84% to 1.13%, and the cookies became progressively harder at higher SBE levels. This most likely arises because the dietary fiber and phenolic compounds introduced with the extract interact with the starch and lipids of the dough, possibly altering water distribution and the structure of the baked matrix, thereby increasing hardness [20]. More specifically, the bran-derived dietary fiber competed for water and partially replaced the low-gluten flour, lowering the final moisture and weakening the continuous starch–protein network, while the SBE phenolics may interact with starch and protein, further stiffening the matrix [1,20]. Overall, these modest changes in color and hardness are comparable to those reported for other phenolic-rich bakery products; however, their effect on consumer acceptance remains to be confirmed by sensory evaluation with a trained panel. The increase in hardness reached statistical significance only at the 2% level (versus the control), whereas fracturability did not differ significantly among the formulations (Table 3).

### 3.3. Phenolic Contents and Antioxidant Stability of Taosu During Storage

The contents of bioactive compounds in Taosu increased substantially with the SBE level at each storage time and declined progressively over 14 days of storage (Table 4). On day 1, the TPC increased from 0.365 mg GAE/g in the control to 1.370 mg GAE/g at the 2% level, and the TFC and TAC followed the same dose-dependent pattern. By day 14, all three indices had decreased, yet the SBE-fortified samples retained markedly higher levels than the control (e.g., 0.530 vs. 0.266 mg GAE/g for TPC at 2% and 0%). Thus, although baking and storage inevitably caused losses of phenolics and anthocyanins, SBE provided a dose-dependent reservoir that maintained higher residual levels of bioactive compounds throughout storage. The cookies kept a perceptibly reddish color over the 14 days of storage (Figure 1). Moreover, the ethanolic extracts prepared from the cookies for the phenolic and antioxidant assays were themselves visibly red, and this color persisted after baking and across the 14 days, fading only slightly while remaining strongly dose-dependent (Figure 1); the depth of the extract color thus paralleled the measured total phenolic, flavonoid, and anthocyanin contents and antioxidant capacity, both across SBE levels and during storage, providing direct visual correlation of these quantitative trends.

The antioxidant capacity showed the same trend (Table 4), as the ABTS, DPPH, and FRAP values increased with the SBE level and decreased during storage, but it remained significantly higher in the SBE-fortified samples than in the control at every time point.

Overall, these results suggest that SBE improved the antioxidant stability of Taosu during storage, mainly through the SBE-derived phenolics retained in the cookies. As shown in Figure 2 and Table 2, the main retained phenolics are likely to include the flavones apigenin and luteolin, the flavonol kaempferol, caffeoylquinic acid, and apigeninidin, all of which are well-documented radical scavengers. The gradual decline of these compounds and of the antioxidant capacity during storage may reflect the progressive oxidation, polymerization, and matrix-binding of phenolics in low-moisture, high-fat baked products, whereas the larger initial pool supplied by SBE likely buffered this loss, keeping the fortified cookies more active throughout storage [29,30]. A similar antioxidant-protective effect of sorghum bran has been documented during the cold storage of beef sausages [31], and keeping a high level of antioxidants is an effective way to slow oxidative deterioration in low-moisture, high-fat bakery products [3,5]. Notably, this protective effect persisted beyond the fresh baseline of the control: even after 14 days of storage, the 2% cookies retained higher total phenolic and anthocyanin contents and stronger DPPH and ABTS radical-scavenging capacity than the freshly baked (day 1) control, so that the two-week-old fortified Taosu remained more antioxidant-active than the unfortified product at its freshest. Such sustained antioxidant protection is the basis on which phenolic-rich natural extracts have been reported to retard oxidative deterioration and prolong the storage stability of lipid-rich and bakery foods [32,33], suggesting that SBE could support improved retention of its antioxidant status during the period evaluated.

### 3.4. Effect of SBE on Acrylamide and 5-HMF Formation

AA and 5-HMF, the two principal heat-induced contaminants of baked cereal foods, both decreased significantly and dose-dependently with SBE addition (Table 5). AA declined from 128.07 ± 0.42 μg/kg in the control to 88.53 ± 15.48 μg/kg at the 2% level (a 30.9% reduction), whereas 5-HMF decreased from 1158.67 ± 227.09 to 626.00 ± 28.21 μg/kg (a 46.0% reduction), with the strongest mitigation consistently observed at the highest addition level (*p* < 0.05).

These reductions coincided with the dose-dependent enrichment of phenolic and 3-deoxyanthocyanidin compounds in fortified Taosu (Table 2, Figure 2) and with the higher antioxidant capacity retained during storage (Table 4). Polyphenols and anthocyanins have been reported to inhibit the formation of AA and 5-HMF during thermal processing through several complementary routes, including the scavenging of free radicals generated in Maillard cascade, trapping of reactive carbonyl and α-dicarbonyl intermediates, and competition with asparagine and reducing sugars for shared Maillard and caramelization precursors [6,34].

In particular, several phenolics that increased in fortified Taosu, notably the flavones apigenin and luteolin and caffeoylquinic acids, are documented scavengers of free radicals and reactive carbonyl intermediates of the Maillard pathway, providing a compositional basis for the reductions observed here [35,36,37]. Benchmarking SBE against a synthetic antioxidant, such as BHT, would further quantify its relative efficacy and is a worthwhile direction for future work. At the molecular level, the major flavones and phenolic acids carried in from SBE, such as apigenin, luteolin, and caffeoylquinic acids, can trap the reactive α-dicarbonyls glyoxal, methylglyoxal, and 3-deoxyglucosone, which are key intermediates of both the acrylamide and 5-HMF pathways, forming stable adducts that divert these precursors from contaminant-forming routes [38]. It should be noted, however, that flavonoids may exert either inhibitory or promotive effects on acrylamide formation depending on their structure and concentration [39,40]; thus, the consistent, dose-dependent decrease found in this study indicates that the carbonyl- and radical-trapping (inhibitory) routes predominated in this matrix.

A comparable mitigating effect of sorghum bran has been reported in antioxidant-fortified wheat bread, in which sorghum bran extract lowered acrylamide formation [10]; phenolic-rich plant extracts and dietary fibers carrying bound polyphenols have likewise suppressed AA and 5-HMF in biscuits and cookies [11,41]. The progressively stronger suppression at higher SBE levels therefore indicates that the incorporated sorghum bran phenolics act as effective inhibitors of Maillard- and caramelization-derived contaminants in this high-temperature baked, low-moisture matrix—an interpretation further supported by changes in Maillard- and lipid-oxidation-related volatiles described in the following section.

### 3.5. Effects of SBE on Taste and Aroma Profiles (Electronic Tongue and HS-SPME-GC-MS)

**Figure 3:**
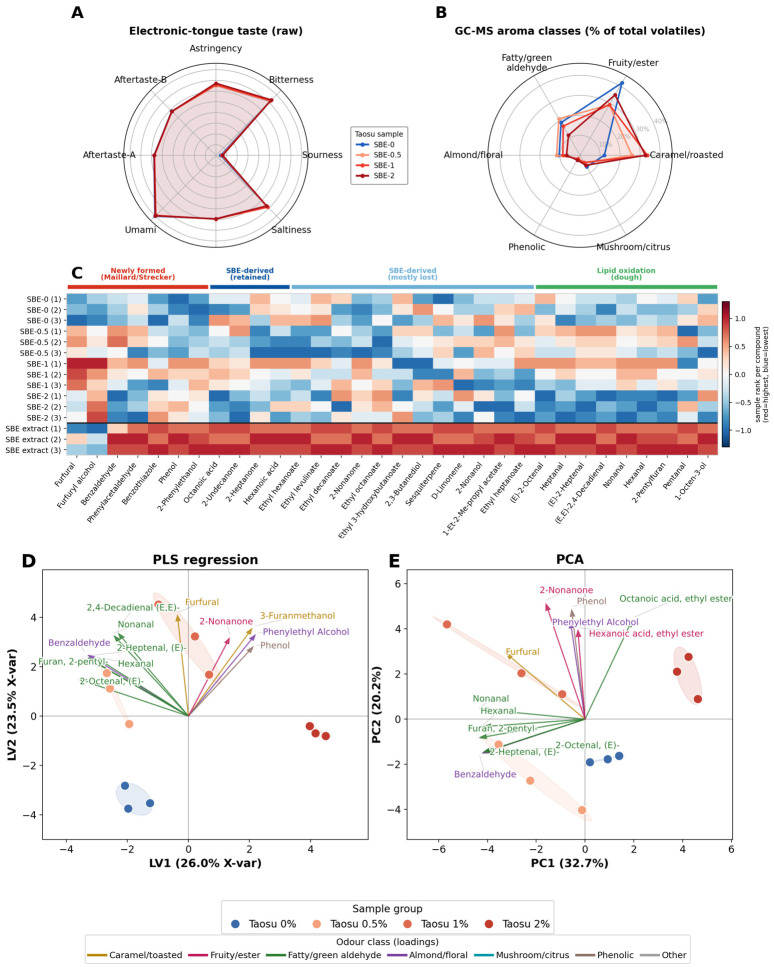
Flavor profiles of Taosu prepared with different SBE levels. (**A**) Electronic-tongue taste attributes (raw sensor response); (**B**) GC-MS aroma classes (% of total volatiles); (**C**) heatmap of the volatile compounds across all individual samples (per-compound sample ranking: red = highest, blue = lowest), with compounds grouped by their fate from the SBE to the cookie; (**D**) PLS regression of the cookie volatile profiles against SBE dose (leave-one-out cross-validated: R^2^Y = 0.98, Q^2^ = 0.73, 1000-permutation *p* = 0.002); (**E**) PCA of the cookie volatile profiles. In (**D**,**E**), the loading arrows and their labels are colored by odor class.

The taste attributes of the four formulations, measured by the electronic tongue across eight indices (sourness, bitterness, astringency, umami, saltiness, richness, and bitter and astringent aftertastes), were highly similar, with their radar profiles overlapping closely (Figure 3A). None of the attributes showed a marked or consistent change with SBE level, indicating that SBE addition did not markedly alter the instrumental taste profile of Taosu at the levels tested. This indicates that the extract can be used to raise the phenolic content of the cookies with only limited changes in the electronic-tongue profile.

The volatile profile of the cookies and the sorghum bran extract was characterized by HS-SPME-GC-MS, which identified 32 aroma compounds, mainly aldehydes, ketones, furans, alcohols, esters, and a few aromatics, each confirmed by NIST23.L spectral matching together with its experimental retention index (RI) (Table 6). The aroma of the control Taosu was dominated by lipid-derived aldehydes (hexanal, nonanal, (E)-2-heptenal, and (E,E)-2,4-decadienal) and methyl ketones together with Maillard-derived furans, a profile typical of a high-fat, short-dough product [24,42]. To capture the sensory character of the volatile fraction, each compound was assigned to one of six odor classes from its characteristic odor descriptors, and the summed relative peak area of each class (% of total identified peak area) was displayed as an aroma radar (Figure 3B). On this basis, the caramel/roasted (Maillard) class expanded from about 12% to 33% of the total volatiles with increasing SBE, whereas the fatty/green-aldehyde (lipid-oxidation) class contracted from about 19% to 12% at the 2% dose, with fruity esters remaining the largest single class throughout [35]. Incorporation of SBE reshaped this profile in a clear dose-dependent manner, as PLS regression against SBE dose captured this trend along its first latent variables and was well-validated by leave-one-out cross-validation (volatiles: R^2^X = 0.64, R^2^Y = 0.98, Q^2^ = 0.73; phenolics: R^2^X = 0.80, R^2^Y = 0.95, Q^2^ = 0.94; 1000-permutation *p* ≤ 0.002) (Figure 3D), while unsupervised PCA of the cookie volatile profiles (Figure 3E) provided an independent cookies-only overview without the extract dominating the ordination.

These changes reflect two routes that are clear at the individual-compound level (Figure 3C, Table 6). SBE strongly promoted Maillard-derived furans, with furfuryl alcohol increasing about ten-fold from the control to 2% SBE and furfural peaking at the intermediate dose [43], whereas the lipid-oxidation aldehydes (hexanal, nonanal, (E)-2-heptenal, and (E,E)-2,4-decadienal) rose at low SBE levels but declined at 2% SBE, with several returning to or below the control. This biphasic behavior of lipid-oxidation aldehydes is consistent with better oxidative stability of SBE cookies, since these volatiles arise from lipid oxidation [35]. Overall, SBE did not simply suppress the Maillard chemistry but reshaped it, as acrylamide and 5-HMF were lowered while Maillard aroma compounds, such as furfuryl alcohol (Figure 3), were retained or even enhanced, consistent with the view that antioxidant interventions can steer the Maillard network away from its toxic products while preserving sensorially important ones [44]. Mechanistically, the main phenolics that SBE introduced into the cookies, namely apigenin, luteolin, the caffeoylquinic acids, and apigeninidin (Figure 2, Table 2), are recognized radical scavengers and may therefore help to limit lipid oxidation [45], such that the radical-scavenging phenolics introduced by SBE curb the generation of hexanal, nonanal, and (E,E)-2,4-decadienal at the highest dose. The aromatic carbonyls, benzaldehyde and phenylacetaldehyde, and alcohol 2-phenylethanol, which the fate analysis assigned to in-cookie formation, instead arise through amino-acid (Strecker-type) routes that are relatively independent of lipid radicals [46]. Together with the reduced acrylamide and 5-HMF, these aroma changes are broadly consistent at the volatile level, with the improved oxidative and thermal stability inferred from the phenolic, antioxidant, and Maillard marker data. Taken together, the electronic tongue and HS-SPME-GC-MS results indicate that SBE measurably adjusts both the taste (mainly more astringent and bitter) and volatile profile (most clearly a shift toward Maillard-derived furans and a suppression of lipid-oxidation aldehydes at the highest dose) of Taosu, while the characteristic baked, sweet, and fatty sensory identity of the product appears to be largely preserved at the levels tested. The odor activity values of these volatiles are given in Table S2.

### 3.6. Correlation Analysis and Integrated Quality Evaluation

**Figure 4:**
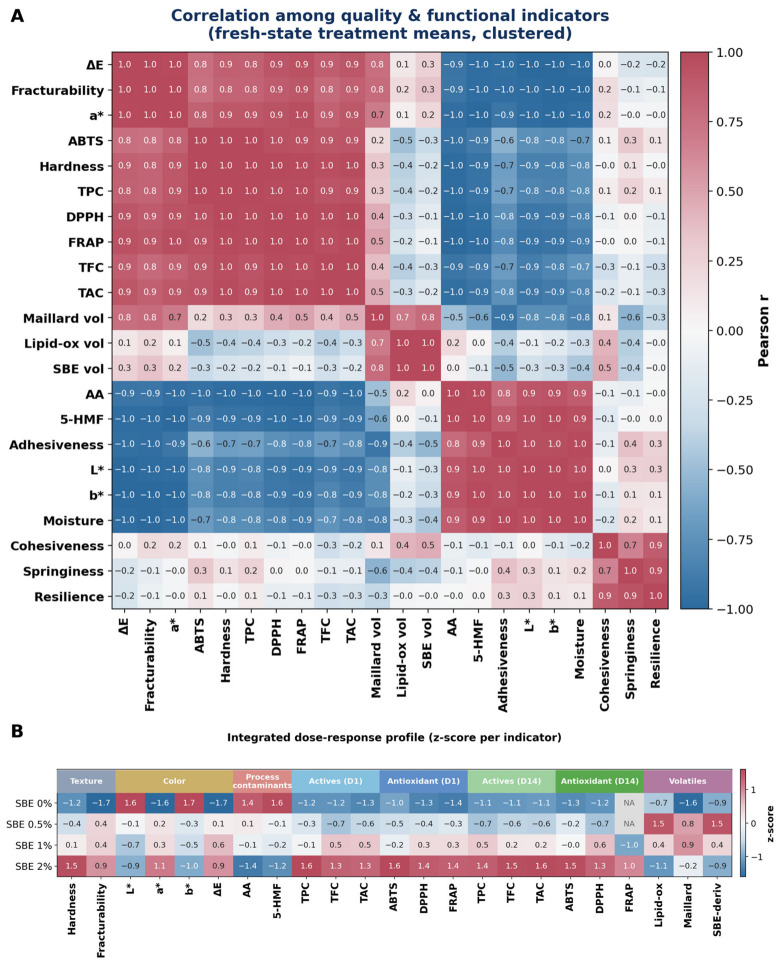
Integrated quality evaluation of Taosu prepared with different SBE levels. (**A**) Pearson correlation matrix between the color, texture, moisture, bioactive (TPC, TFC, TAC), antioxidant (DPPH, FRAP, ABTS), heat-contaminant (acrylamide, 5-HMF) and volatile-class (lipid oxidation, Maillard/Strecker, and SBE-derived) indicators (fresh-state treatment means, ordered by hierarchical clustering). (**B**) Z-scored dose–response heatmap of the same indicators, including the day 1 and day 14 storage values for the bioactive and antioxidant variables, across SBE levels.

To evaluate how the different quality attributes of Taosu responded to SBE in a coordinated way, the color, texture, moisture, bioactive, antioxidant, and heat-contaminant indicators were examined together by Pearson correlation (Figure 4A); the full correlation matrix for all individual parameters is provided in Supplementary Table S6. The variables were separated into two blocks that were strongly and inversely related (as associations among the treatment means). The first grouped the total phenolic, flavonoid, and anthocyanin contents (TPC, TFC, TAC); the three antioxidant indices (DPPH, FRAP, ABTS); redness (a*); and hardness, all of which rose with SBE level; conversely, the second grouped acrylamide, 5-HMF, lightness (L*), yellowness (b*), and moisture, which fell as SBE increased. This inverse structure is consistent with SBE phenolic enrichment acting as a common contributor to these coordinated changes, as phenolics can raise antioxidant capacity through radical scavenging and may lower acrylamide and 5-HMF through scavenging of Maillard radicals and trapping of reactive carbonyls. The very strong phenolic-antioxidant correlation (for example, TPC and DPPH, r = 0.98), which also held at the replicate level in the storage data set, and the equally strong phenolic-contaminant anticorrelation (TPC and AA, r = −0.98) therefore support this phenolic-related interpretation without establishing causality.

The z-scored heatmap (Figure 4B) translated this structure into a dose ranking: the control occupied the least favorable position on almost every axis (lowest bioactive and antioxidant values, highest acrylamide and 5-HMF), the 0.5% and 1% formulations were intermediate, and the 2% formulation gave the most favorable overall profile. These coordinated gains are consistent with a single group of constituents contributing to several roles at once, as SBE phenolics can act as antioxidants (raising DPPH, FRAP, and ABTS), potential inhibitors of Maillard- and caramelization-derived contaminants (lowering acrylamide and 5-HMF), and natural colorants (increasing redness); this multifunctionality distinguishes SBE from additives that improve only a single attribute. The capacity of a single natural extract to improve bioactive content, antioxidant capacity and thermal-processing safety at the same time has also been reported for other phenolic-rich plant materials added to bakery products [8,17], and the present results show that SBE behaves similarly in a traditional Chinese shortbread.

These gains were accompanied by the expected trade-offs shown in the same analysis; higher SBE darkened the cookies and tended to increase hardness, and it shifted the volatile fraction (Figure 3), although the instrumental taste profile was largely unchanged. Within the range studied, the 2% level offered the best overall balance of bioactive, antioxidant and processing-safety quality, with the accompanying color and textural changes remaining within an acceptable range for this product type.

## 4. Conclusions

This study shows that SBE, obtained from an abundant cereal by-product, can act as a single, multifunctional, and clean-label ingredient for traditional Chinese baked goods. Incorporated into Taosu, SBE simultaneously enriched cookies with phenolic and anthocyanin antioxidants, suppressed heat-induced contaminants acrylamide and 5-HMF, and imparted a natural reddish color, all while leaving the instrumental taste profile essentially unchanged and steering the volatile profile toward Maillard caramel notes. Correlation and integrated analysis suggested that these benefits are associated with coordinated phenolic-driven changes rather than isolated effects, supporting the potential of SBE as a multifunctional ingredient for Taosu.

Beyond raising the initial quality, SBE also sustained the antioxidant status of Taosu throughout storage, indicating improved retention of its antioxidant status during the period evaluated. This upcycled milling by-product was also associated with lower levels of acrylamide and 5-hydroxymethylfurfural, as well as lower lipid-oxidation aldehydes at the highest SBE level. Taken together, these findings position sorghum bran as a promising route for valorizing a low-value milling by-product into a natural functional ingredient with potential to improve the antioxidant value and processing-safety profile of traditional bakery products. Future work could focus on optimizing the baking process to maximize these benefits and on sensory and consumer-acceptance studies to confirm the market potential of the fortified Taosu.

## Figures and Tables

**Table 1:** Formulation of Taosu samples with different levels of SBE addition.

Taosu Sample	Unsalted Butter (g)	Corn Oil (g)	Sodium Bicarbonate (g)	Baking Powder (g)	Powdered Sugar (g)	Low-Gluten Wheat Flour (g)	SBE (g)
SBE-0	30	45	1.2	1.5	40	125.00	0.00
SBE-0.5	30	45	1.2	1.5	40	124.16	0.84
SBE-1	30	45	1.2	1.5	40	123.32	1.68
SBE-2	30	45	1.2	1.5	40	121.65	3.35

SBE addition levels (0, 0.5, 1.0, and 2.0%) are expressed as a percentage of the total dry powder, with SBE replacing an equal mass of low-gluten wheat flour while the amounts of the other ingredients are kept constant.

**Table 2:** Phenolic compounds tentatively identified in SBE and Taosu by UHPLC-QTOF-MS/MS.

No	Compound	Class	Ion	Adduct	RT	*m*/*z*	Error (ppm)	Key MS/MS Fragments
1	Pelargonin isomer 1	Anthocyanins	POS	[M]+	9.68	595.1656	−0.2	271.06014, 433.11376, 595.16314, 153.02064
2	Cyanidin-3,5-di-O-glucoside isomer 1	Anthocyanins	POS	[M]+	9.98	611.1623	2.7	287.05522, 449.10920, 303.05232, 355.06637
3	Apigeninidin	Anthocyanins	Both	[M + H]+	10.08	255.0657	2.1	255.06600, 171.04401, 157.06546, 128.06085
4	Peonidin-3-O-glucoside	Anthocyanins	NEG	[M − 2H]−	12.36	461.107	−3.1	299.05568, 461.10860, 284.03153, 141.86873
5	Cyanidin-3,5-di-O-glucoside isomer 2	Anthocyanins	POS	[M]+	12.38	611.163	3.8	287.05479, 611.16140, 145.05054, 611.18194
6	Pelargonin isomer 2	Anthocyanins	POS	[M]+	12.89	595.1652	−0.9	271.05985, 595.16472, 595.11288, 266.99635
7	Malvidin-3-O-glucoside	Anthocyanins	NEG	[M − 2H]−	16.93	491.1182	−1.6	329.06652, 314.04287, 491.11994, 313.03260
8	Flavone (unspecified) isomer 1	Flavones	Both	[M − H]−	9.97	609.1451	−1.7	609.14677, 285.03988, 283.02341, 446.08512
9	Isoshaftoside	Flavones	POS	[M + H]+	10.43	565.1548	−2.2	379.08028, 397.09221, 325.07427, 511.12278
10	Isovitexin	Flavones	NEG	[M − H]−	11.24	431.0974	−2.3	311.05505, 283.05985, 431.10014, 341.06382
11	Vitexin	Flavones	POS	[M + H]+	11.25	433.1123	−1.5	313.07075, 283.05957, 397.09367, 323.09080
12	Tectochrysin	Flavones	NEG	[M − H]−	11.37	267.0662	−0.3	180.05703, 224.04689, 252.04245, 267.06519
13	7-O-Methylchrysin	Flavones	POS	[M + H]+	11.52	269.0814	5.3	254.05804, 269.08105, 226.06203, 157.06427
14	Cynaroside isomer 1	Flavones	NEG	[M − H]−	11.74	447.0926	−1.6	285.04071, 284.03249, 447.09136, 203.03416
15	Glucoluteolin	Flavones	POS	[M + H]+	11.74	449.1075	−5.5	287.05452, 255.06302, 449.11987, 389.09187
16	Apigenin-7-O-glucoside isomer 1	Flavones	POS	[M + H]+	11.90	433.1122	−1.6	271.06018, 153.01793, 98.98248, 85.02838
17	Flavone (unspecified) isomer 2	Flavones	NEG	[M − H]−	12.36	609.1454	−1.2	609.14720, 285.03988, 284.03223, 429.08250
18	Diosmetin	Flavones	NEG	[M − H]−	12.44	299.0558	−0.9	133.02980, 283.02451, 284.03260, 299.05433
19	Chrysoeriol	Flavones	POS	[M + H]+	12.45	301.0711	1.3	301.07204, 286.04850, 258.05279, 285.03901
20	Rhoifolin	Flavones	Both	[M − H]−	12.58	577.1552	−1.7	269.04561, 577.15538, 268.03797, 531.16434
21	Plantaginin	Flavones	NEG	[M − H]−	12.70	447.0917	−3.6	284.03257, 285.04064, 447.09190, 136.98706
22	Neodiosmin	Flavones	NEG	[M − H]−	12.86	607.1653	−2.6	299.05602, 284.03274, 607.16906, 607.13216
23	Diosmin	Flavones	POS	[M + H]+	12.87	609.1805	−1.5	301.07012, 463.12414, 609.18705, 85.02766
24	Apigenin-7-O-glucoside isomer 2	Flavones	Both	[M − H]−	13.07	431.0974	−2.2	268.03830, 431.09767, 269.04441, 240.03900
25	7-Glu Chrysoeriol	Flavones	NEG	[M − H]−	13.23	461.1076	−2.8	283.02413, 461.10634, 446.08198, 299.05594
26	5,7-Dihydroxy-2′-methoxyflavone	Flavones	NEG	[M − H]−	13.86	283.0618	2.2	240.04349, 268.03861, 283.06167, 267.03083
27	Santin isomer 1	Flavones	POS	[M + H]+	14.25	345.0968	−0.1	345.09739, 329.06620, 330.07393, 284.06889
28	Velutin	Flavones	POS	[M + H]+	14.30	315.0871	2.5	300.06449, 315.08840, 272.06910, 229.05056
29	Luteolin-7-glucoside	Flavones	POS	[M + H]+	14.43	449.1075	−0.8	287.05579, 449.10759, 418.18192, 121.03091
30	Cynaroside isomer 2	Flavones	NEG	[M − H]−	14.44	447.0918	−3.4	285.03998, 284.03226, 447.09212, 241.04889
31	Pectolinarin	Flavones	POS	[M + H]+	16.08	623.1958	−2	315.08614, 477.14084, 300.06509, 623.15570
32	Luteolin	Flavones	Both	[M − H]−	16.10	285.0409	1.5	285.04103, 133.03007, 151.00397, 175.04030
33	Nepetin	Flavones	POS	[M + H]+	16.29	317.067	4.4	302.04250, 168.00493, 317.06439, 140.01052
34	6-Methoxyluteolin	Flavones	NEG	[M − H]−	16.30	315.0507	−1.2	300.02846, 136.98788, 315.04897, 299.02001
35	Methoxyflavone O-hexoside (unspecified)	Flavones	POS	[M + H]+	16.91	493.133	−2	331.08129, 316.05727, 493.13541, 329.06959
36	3′,4′-Dihydroxyflavone	Flavones	NEG	[M − H]−	17.38	253.0498	−3.3	133.03035, 253.05210, 225.05288, 129.03314
37	Apigenin	Flavones	Both	[M − H]−	18.05	269.0458	0.9	117.03607, 269.04907, 151.00410, 149.02473
38	Hispidulin	Flavones	NEG	[M − H]−	18.27	299.0564	0.8	284.03357, 136.98845, 65.00418, 283.02538
39	Methoxyflavone (unspecified isomer)	Flavones	POS	[M + H]+	18.27	301.0715	2.8	286.05061, 301.07154, 168.00534, 140.01007
40	Tricin	Flavones	Both	[M − H]−	18.32	329.066	−2	271.02540, 299.01890, 314.04120, 227.03502
41	Jaceosidin	Flavones	POS	[M + H]+	18.71	331.082	0.1	316.05876, 331.08142, 301.03549, 273.03953
42	4′,5,7-trihydroxy-3,6-dimethoxyflavone	Flavones	NEG	[M − H]−	18.71	329.0662	−1.4	299.02056, 314.04506, 271.02588, 199.04031
43	5,4′-Dihydroxy-7-methoxyflavone	Flavones	NEG	[M − H]−	18.89	283.0604	−2.8	240.04307, 268.03399, 239.03434, 159.00564
44	Cirsimaritin isomer 1	Flavones	Both	[M + H]+	20.24	315.0866	0.9	254.05778, 315.08686, 282.05261, 136.01575
45	Santin isomer 2	Flavones	POS	[M + H]+	20.53	345.0968	−0.3	330.07367, 345.09679, 169.01239, 168.00483
46	Eupatilin	Flavones	NEG	[M − H]−	20.53	343.0814	−2.9	313.03526, 298.01132, 328.05924, 270.01570
47	Acacetin	Flavones	Both	[M − H]−	21.64	283.0611	−0.3	268.03809, 283.06101, 239.03498, 117.03390
48	Dimethoxyflavone (unspecified isomer)	Flavones	POS	[M + H]+	21.74	315.0877	−7.4	300.06418, 168.00533, 315.08722, 140.00941
49	Cirsimaritin isomer 2	Flavones	NEG	[M − H]−	21.74	313.0717	0	283.02503, 298.04814, 255.03011, 163.00400
50	7-Hydroxy-3′-methoxyflavone	Flavones	NEG	[M − H]−	22.16	267.0656	−2.4	267.06953, 180.05786, 224.04756, 252.04160
51	7,4′-Di-O-methylapigenin	Flavones	POS	[M + H]+	24.68	299.091	3.3	299.09230, 256.07325, 284.06796, 167.03380
52	Naringenin-7-O-glucoside	Flavanones	NEG	[M − H]−	11.16	433.1117	−5.4	271.05870, 151.00537, 313.05106, 119.05151
53	Sternbin	Flavanones	NEG	[M − H]−	11.83	301.0708	−3.1	135.04715, 139.04053, 124.01972, 165.01693
54	Sakuranetin	Flavanones	NEG	[M − H]−	13.54	285.0768	−0.3	119.05013, 165.01900, 65.00344, 97.03006
55	Eriodictyol	Flavanones	Both	[M − H]−	15.61	287.0559	−0.8	135.04562, 151.00340, 107.01293, 134.03901
56	4′-Hydroxy-5,7-dimethoxyflavanone	Flavanones	POS	[M + H]+	15.70	301.1068	−0.9	181.04881, 138.03425, 147.04549, 166.02651
57	Flavanone (unspecified isomer)	Flavanones	POS	[M + H]+	16.95	241.0855	−1.9	121.02800, 147.04336, 119.04907, 91.05365
58	2′-Hydroxyflavanone	Flavanones	NEG	[M − H]−	16.96	239.0708	−2.5	119.05099, 145.03019, 197.05880, 239.07052
59	Naringenin	Flavanones	Both	[M − H]−	17.82	271.0618	2.1	119.05032, 151.00389, 107.01386, 65.00373
60	5,7,4′-Trihydroxy-8-methylflavanone	Flavanones	POS	[M + H]+	19.88	287.0906	2.1	167.03318, 124.01609, 152.01016, 119.04898
61	Kaempferol-7-O-neohesperidoside	Flavonols	NEG	[M − H]−	11.40	593.1508	−0.6	285.03927, 593.14847, 593.07380, 199.04395
62	Demethoxycentaureidin 7-O-rutinoside	Flavonols	NEG	[M − H]−	13.53	637.1771	−0.6	329.06540, 637.17587, 314.04349, 313.03115
63	Robinetin trimethyl ether	Flavonols	NEG	[M − H]−	14.25	343.081	−3.9	313.03147, 328.05674, 285.03968, 343.08276
64	Kaempferol isomer 1	Flavonols	NEG	[M − H]−	14.48	285.0408	1.1	285.04142, 117.03523, 137.02464, 119.05090
65	3-Hydroxy-3′,4′-dimethoxyflavone	Flavonols	POS	[M + H]+	17.91	299.0912	−0.8	299.09149, 284.06831, 255.06505, 283.05995
66	Kaempferol isomer 2	Flavonols	Both	[M − H]−	18.05	285.0406	0.6	285.04134, 133.02987, 117.03560, 165.01988
67	Kaempferide	Flavonols	NEG	[M − H]−	19.57	299.0554	−2.3	284.03284, 163.00534, 164.01207, 299.05277
68	Demethyltexasin	Isoflavones	POS	[M + H]+	13.08	271.0595	−5.4	271.06121, 145.03096, 197.05917, 225.05915
69	Tectorigenin	Isoflavones	NEG	[M − H]−	13.14	299.0553	−2.8	284.03059, 283.02082, 110.00117, 165.99204
70	Genistin	Isoflavones	NEG	[M − H]−	13.19	431.0976	−1.7	268.03767, 269.04464, 431.09796, 311.05720
71	Daidzein	Isoflavones	Both	[M − H]−	15.00	253.0508	0.7	253.05033, 224.04793, 133.02967, 208.05243
72	Glycitein	Isoflavones	Both	[M + H]+	15.53	285.0756	−1.5	285.07626, 270.05322, 242.05818, 229.08706
73	Biochanin A isomer 1	Isoflavones	Both	[M + H]+	16.58	285.0758	0.4	285.07632, 269.04477, 270.05285, 242.05893
74	Biochanin A isomer 2	Isoflavones	NEG	[M − H]−	22.62	283.0606	−2.1	268.03664, 239.03370, 240.04308, 211.04029
75	Neochlorogenic acid	Hydroxycinnamic acids	NEG	[M − H]−	6.05	353.0878	−0.1	191.05633, 135.04512, 179.03519, 134.03829
76	Quinic acid	Hydroxycinnamic acids	NEG	[M − H]−	7.60	353.0879	4.8	191.05646, 85.02987, 127.03968, 111.04666
77	Feruloylquinic acid	Hydroxycinnamic acids	NEG	[M − H]−	8.04	367.1019	−4.3	134.03640, 193.04931, 135.04884, 149.05981
78	Caffeoylquinic acid isomer 1	Hydroxycinnamic acids	Both	[M − H]−	8.06	353.0879	0.2	173.04523, 135.04531, 191.05584, 179.03506
79	3-(3-Hydroxyphenyl)propionic acid	Hydroxycinnamic acids	NEG	[M − H]−	9.31	165.0562	2.9	59.01438, 93.03455, 119.05125, 121.06700
80	4-coumarate	Hydroxycinnamic acids	NEG	[M − H]−	9.96	163.04	−0.1	119.05049, 93.03548, 117.03572, 91.05628
81	Dihydroferulic acid	Hydroxycinnamic acids	NEG	[M − H]−	10.20	195.067	3.8	121.03006, 136.05314, 135.04522, 93.03476
82	Sinapic acid	Hydroxycinnamic acids	POS	[M + H]+	11.04	225.0751	−2.8	91.05401, 119.04858, 147.04383, 65.03879
83	Caffeoylquinic acid isomer 2	Hydroxycinnamic acids	Both	[M − H]−	13.62	515.1186	−1.8	173.04535, 353.08658, 179.03521, 191.05683
84	3-Hydroxy-4-methoxycinnamic acid	Hydroxycinnamic acids	NEG	[M − H]−	16.04	193.0509	1.6	133.02832, 133.03732, 134.03762, 132.02056
85	Diferuloyl glycerol	Hydroxycinnamic acids	NEG	[M − H]−	19.25	443.1331	−3.7	193.05082, 134.03802, 443.13515, 160.01650
86	Resveratrol isomer 1	Stilbenes	NEG	[M − H]−	14.33	227.071	−1.7	143.05034, 227.06850, 92.99594, 185.06340
87	Resveratrol isomer 2	Stilbenes	NEG	[M − H]−	20.61	227.071	−1.8	185.06200, 227.06753, 117.03476, 156.05544
88	Pterostilbene isomer 1	Stilbenes	Both	[M + H]+	22.76	257.1169	−1.3	181.06548, 153.07145, 165.07113, 242.09819
89	Pterostilbene isomer 2	Stilbenes	POS	[M + H]+	23.02	257.1169	−1.2	181.06762, 165.07106, 121.06220, 242.09351

Tentative identification (database matching of accurate mass and MS/MS); full peak-area data are provided in Table S1 (Supplementary Materials).

**Table 3:** Color, moisture, and textural properties of Taosu with different levels of SBE.

Parameter	SBE-0	SBE-0.5	SBE-1	SBE-2
Moisture (%)	1.840 ± 0.134 ^a^	1.213 ± 0.211 ^b^	1.218 ± 0.131 ^b^	1.127 ± 0.174 ^b^
L*	46.31 ± 1.44 ^a^	34.27 ± 0.70 ^b^	29.77 ± 0.99 ^c^	28.28 ± 0.97 ^d^
a*	5.14 ± 0.51 ^c^	9.84 ± 0.70 ^b^	10.06 ± 1.04 ^b^	12.20 ± 0.63 ^a^
b*	18.93 ± 1.75 ^a^	12.03 ± 0.47 ^b^	11.41 ± 0.66 ^b^	9.64 ± 0.76 ^c^
ΔE	0.00	14.67 ± 0.59 ^c^	18.87 ± 1.00 ^b^	21.50 ± 0.88 ^a^
Hardness (g)	26,934 ± 19,507 ^b^	31,969 ± 13,452 ^ab^	35,744 ± 5120 ^ab^	45,302 ± 6592 ^a^
Fracturability (g)	8793 ± 9578 ^a^	18,708 ± 15,627 ^a^	18,875 ± 11,165 ^a^	21,379 ± 13,077 ^a^
Adhesiveness (g·s)	−64 ± 40 ^a^	−102 ± 23 ^ab^	−106 ± 26 ^b^	−102 ± 23 ^ab^
Springiness	0.263 ± 0.168 ^a^	0.260 ± 0.085 ^a^	0.228 ± 0.034 ^a^	0.271 ± 0.037 ^a^
Cohesiveness	0.354 ± 0.044 ^ab^	0.412 ± 0.076 ^a^	0.323 ± 0.046 ^b^	0.371 ± 0.044 ^ab^
Resilience	0.257 ± 0.034 ^a^	0.268 ± 0.090 ^a^	0.220 ± 0.032 ^a^	0.262 ± 0.032 ^a^

Values are mean ± SD; different lowercase letters in rows indicate significant differences among SBE levels (*p* < 0.05).

**Table 4:** Bioactive compound contents and antioxidant capacity of Taosu during storage.

Index	Storage	SBE-0	SBE-0.5	SBE-1	SBE-2
TPC (mg GAE/g)	Day 1	0.365 ± 0.019 ^Ad^	0.681 ± 0.007 ^Ac^	0.771 ± 0.004 ^Ab^	1.370 ± 0.028 ^Aa^
	Day 7	0.339 ± 0.003 ^Bd^	0.485 ± 0.004 ^Bc^	0.619 ± 0.010 ^Bb^	0.838 ± 0.002 ^Ba^
	Day 14	0.266 ± 0.005 ^Cd^	0.310 ± 0.011 ^Cc^	0.438 ± 0.004 ^Cb^	0.530 ± 0.004 ^Ca^
TFC (mg RE/g)	Day 1	2.964 ± 0.133 ^Ac^	3.512 ± 0.183 ^Ac^	5.097 ± 0.259 ^Ab^	6.089 ± 0.321 ^Aa^
	Day 7	1.154 ± 0.185 ^Bc^	1.306 ± 0.090 ^Bc^	1.689 ± 0.126 ^Bb^	2.643 ± 0.153 ^Ba^
	Day 14	0.487 ± 0.187 ^Cc^	0.705 ± 0.228 ^Cc^	0.999 ± 0.107 ^Cb^	1.523 ± 0.199 ^Ca^
TAC (mg LutE/g)	Day 1	0.677 ± 0.022 ^Ad^	0.852 ± 0.034 ^Ac^	1.107 ± 0.034 ^Ab^	1.292 ± 0.042 ^Aa^
	Day 7	0.637 ± 0.008 ^Ad^	0.814 ± 0.007 ^Ac^	0.867 ± 0.008 ^Bb^	0.996 ± 0.007 ^Ba^
	Day 14	0.509 ± 0.017 ^Bd^	0.560 ± 0.009 ^Bc^	0.639 ± 0.020 ^Cb^	0.784 ± 0.009 ^Ca^
ABTS (mg TE/g)	Day 1	0.419 ± 0.008 ^Ac^	0.464 ± 0.017 ^Ab^	0.487 ± 0.010 ^Ab^	0.632 ± 0.006 ^Aa^
	Day 7	0.255 ± 0.006 ^Bd^	0.331 ± 0.008 ^Bc^	0.362 ± 0.007 ^Bb^	0.510 ± 0.010 ^Ba^
	Day 14	0.208 ± 0.013 ^Cc^	0.311 ± 0.012 ^Bb^	0.334 ± 0.007 ^Cb^	0.492 ± 0.007 ^Ba^
DPPH (mg TE/g)	Day 1	0.205 ± 0.013 ^Ad^	0.337 ± 0.012 ^Ac^	0.436 ± 0.016 ^Ab^	0.591 ± 0.011 ^Aa^
	Day 7	0.175 ± 0.008 ^Bc^	0.204 ± 0.005 ^Bc^	0.297 ± 0.026 ^Bb^	0.392 ± 0.018 ^Ba^
	Day 14	0.149 ± 0.002 ^Cc^	0.170 ± 0.003 ^Cc^	0.233 ± 0.011 ^Cb^	0.269 ± 0.013 ^Ca^
FRAP (mg TE/g)	Day 1	0.123 ± 0.023 ^Ad^	0.268 ± 0.035 ^Ac^	0.345 ± 0.017 ^Ab^	0.482 ± 0.016 ^Aa^
	Day 7	0.100 ± 0.008 ^Ad^	0.168 ± 0.024 ^Bc^	0.301 ± 0.006 ^Bb^	0.344 ± 0.016 ^Ba^
	Day 14	NA	NA	0.031 ± 0.007 ^Cb^	0.115 ± 0.024 ^Ca^

Values are mean ± SD. Uppercase letters indicate differences among storage times; lowercase letters indicate differences among SBE levels (*p* < 0.05). NA, not available.

**Table 5:** Acrylamide (AA) and 5-HMF contents of Taosu with different levels of SBE.

Sample	AA (μg/kg)	5-HMF (μg/kg)
SBE-0	128.07 ± 0.42 ^a^	1158.67 ± 227.09 ^a^
SBE-0.5	110.40 ± 5.10 ^b^	841.33 ± 57.46 ^b^
SBE-1	106.60 ± 6.68 ^b^	814.67 ± 31.90 ^b^
SBE-2	88.53 ± 15.48 ^c^	626.00 ± 28.21 ^c^

Values are mean ± SD; different lowercase letters in a column indicate significant differences (*p* < 0.05).

**Table 6:** Volatile compounds in Taosu and sorghum bran extract (SBE), identified by HS-SPME-GC-MS, and their fate from the extract to the cookie.

No.	Compound	Class	RI (exp)	RI (lit)	ID	Fate (Extract → Taosu)	Odor
1	Pentanal	Aldehyde	735	700	MS, RI	Lipid oxidation (dough)	Pungent/malty/almond
2	Hexanal	Aldehyde	799	800	MS, RI	Lipid oxidation (dough)	Grassy/apple/fatty
3	Heptanal	Aldehyde	900	901	MS, RI	Lipid oxidation (dough)	Fatty/citrus
4	(E)-2-Heptenal	Aldehyde	954	958	MS, RI	Lipid oxidation (dough)	Fatty/grassy/almond
5	Benzaldehyde	Aldehyde	956	961	MS, RI	Newly formed (Maillard)	Bitter almond/cherry
6	Phenylacetaldehyde	Aldehyde	1041	1045	MS, RI	Newly formed (Maillard)	Honey/rose/floral
7	(E)-2-Octenal	Aldehyde	1057	1060	MS, RI	Lipid oxidation (dough)	Fatty/nutty/green
8	Nonanal	Aldehyde	1104	1104	MS, RI	Lipid oxidation (dough)	Fatty/citrus/green
9	(E,E)-2,4-Decadienal	Aldehyde	1316	1317	MS, RI	Lipid oxidation (dough)	Deep-fried/fatty (strong)
10	2-Heptanone	Ketone	889	891	MS, RI	SBE-derived (retained)	Fruity/blue cheese
11	2-Nonanone	Ketone	1092	1091	MS, RI	SBE-derived (mostly lost)	Fruity/floral/warm milk
12	2-Undecanone	Ketone	1294	1294	MS, RI	SBE-derived (retained)	Fruity/orange/waxy
13	2,3-Butanediol	Alcohol	784	785	MS, RI	SBE-derived (mostly lost)	—
14	1-Octen-3-ol	Alcohol	986	980	MS, RI	Lipid oxidation (dough)	Mushroom/earthy
15	2-Nonanol	Alcohol	1100	1100	MS, RI	SBE-derived (mostly lost)	Waxy/citrus
16	2-Phenylethanol	Alcohol	1110	1116	MS, RI	Newly formed (Maillard)	Rose/honey
17	Furfural	Furan	829	831	MS, RI	Newly formed (Maillard)	Almond/caramel/bready
18	Furfuryl alcohol	Furan	851	852	MS, RI	Newly formed (Maillard)	Caramel/bready/sweet
19	2-Pentylfuran	Furan	990	992	MS, RI	Lipid oxidation (dough)	green bean/Buttery/green
20	1-Ethyl-2-methylpropyl acetate	Ester	898	901	MS, RI	SBE-derived (mostly lost)	—
21	Ethyl 3-hydroxybutanoate	Ester	932	945	MS, RI	SBE-derived (mostly lost)	—
22	Ethyl hexanoate	Ester	1000	1000	MS, RI	SBE-derived (mostly lost)	Fruity/pineapple
23	Ethyl levulinate	Ester	1063	1062	MS, RI	SBE-derived (mostly lost)	—
24	Ethyl heptanoate	Ester	1099	1097	MS, RI	SBE-derived (mostly lost)	Fruity
25	Ethyl octanoate	Ester	1199	1196	MS, RI	SBE-derived (mostly lost)	Fruity/waxy/brandy
26	Ethyl decanoate	Ester	1397	1394	MS, RI	SBE-derived (mostly lost)	Fruity/grape
27	Hexanoic acid	Acid	997	990	MS, RI	SBE-derived (retained)	Sweaty/cheese
28	Octanoic acid	Acid	1180	1180	MS, RI	SBE-derived (retained)	Fatty/rancid
29	Phenol	Phenol	981	980	MS, RI	Newly formed (Maillard)	Medicinal/phenolic
30	D-Limonene	Terpene	1025	1031	MS, RI	SBE-derived (mostly lost)	Citrus/lemon
31	Sesquiterpene	Terpene	1528	1490	MS, RI	SBE-derived (mostly lost)	Woody
32	Benzothiazole	N/S-heterocycle	1219	1226	MS, RI	Newly formed (Maillard)	Rubbery/roasted

RI(exp), experimental retention index from C8–C17 n-alkane external standards (Table S3); RI(lit), literature non-polar value (n.a., not available); ID, NIST23.L reverse match factor ≥ 70 with retention-index confirmation (|ΔRI| ≤ 20 where authentic standards or the reliable literature retention indices were available). Relative contents for each formulation and full µg/kg semi-quantification are given in Supplementary Table S2.

## Data Availability

The data presented in this study are available within the article and its Supplementary Materials.

## Supplementary Materials

The following supporting information can be downloaded at: https://www.mdpi.com/article/10.3390/foods15142444/s1, Table S1. Phenolic and related compounds in the SBE and Taosu by UHPLC-QTOF-MS/MS (complete data, with peak areas); Table S2. Volatile compounds of Taosu (SBE-0–SBE-2) and the SBE extract by HS-SPME-GC-MS: identification, fate, odor, semi-quantification (3-octanone equivalents, µg/kg; mean ± SD) and odor activity values (OAV); Table S3. n-Alkane (C8–C17) external-standard retention-index calibration (R^2^ = 0.9994); Table S4. Calibration parameters of the six spectrophotometric assays (TPC, TFC, TAC, DPPH, ABTS and FRAP); Table S5. Validation parameters of the acrylamide and 5-hydroxymethylfurfural LC-MS/MS method (stable-isotope-dilution); Table S6. Comprehensive Pearson correlation matrix among all measured Taosu indicators—quality, antioxidant capacity, processing-safety contaminants, every identified phenolic compound (prefix “P:”) and every volatile compound (prefix “V:”)—across the four SBE formulations (SBE-0, SBE-0.5, SBE-1, SBE-2).

## Abbreviations

The following abbreviations are used in this manuscript:

SBE	Sorghum bran extract
3-DXA	3-Deoxyanthocyanidin
AA	Acrylamide
5-HMF	5-Hydroxymethylfurfural
TPC	Total phenolic content
TFC	Total flavonoid content
TAC	Total anthocyanin content
DPPH	2,2-Diphenyl-1-picrylhydrazyl
ABTS	2,2′-Azino-bis(3-ethylbenzothiazoline-6-sulfonic acid)
FRAP	Ferric reducing antioxidant power
GAE	Gallic acid equivalent
PCA	Principal component analysis
